# Preliminary Biometric Study on Symmetry of Hoof Solear Aspect in Forelimbs in Four Horse Breeds

**DOI:** 10.3390/ani15233369

**Published:** 2025-11-21

**Authors:** Anna Stachurska, Elżbieta Wnuk, Jarosław Łuszczyński, Weronika Donderowicz

**Affiliations:** 1 Department of Horse Breeding and Use, Faculty of Animal Sciences and Bioeconomy, University of Life Sciences in Lublin, 13 Akademicka Street, 20-950 Lublin, Poland; anna.stachurska@up.lublin.pl (A.S.);; 2 Department of Genetics, Animal Breeding and Ethology, Faculty of Animal Science, University of Agriculture in Cracow, 30-059 Cracow, Poland; jaroslaw.luszczynski@urk.edu.pl

**Keywords:** horse, hoof, forelimb, solear aspect, asymmetry, dimension, sex, breed, age

## Abstract

Correct conformation and symmetry of bilateral hooves are associated with a properly balanced hoof, which has a significant impact on the biomechanics of movement and reduces the risk of injury. The aim of the study was to compare solear aspect dimensions of bilateral hooves in forelimbs (hoof width, length, frog width, length, medial and lateral diagonals) with regard to sex, breed and age of horses. The dimensions were measured with a calliper in 100 horses of four breeds. The results showed that the breed factor significantly affected all dimensions. Age was significant for some variables, while sex was not a significant factor. The hoof solear dimensions did not differ between mares and geldings, decreased with a corresponding decrease in horse breed size and often increased with age. Significant differences in the mean dimensions between bilateral hooves were rare and did not show a clear tendency across the population. However, considering the prevalence, almost 1/3 of horses showed asymmetries in particular dimensions. It was concluded that there are no consistent, one-directional asymmetries in the solear aspect of bilateral hooves in horse forelimbs despite frequent two-directional asymmetries occurring in horses. Trimmers should regard the findings to try to maintain hoof symmetry.

## Simple Summary

Morphological features of the foot and the relationship between the limb posture and hoof conformation are crucial for proper gait and may enhance injury prevention by precise trimming and therapeutic horseshoeing. Any defects in hoof shape may result in uneven distribution of forces, which leads to overloading specific areas of the hoof. However, asymmetrical hooves sometimes occur in horses. The current study investigated whether there are any consistent differences between the dimensions of the solear aspect of the right and left hooves in the forelimbs of horses of different sexes, breeds, and ages. Six forelimb hoof dimensions were measured in one hundred horses: hoof width and length, frog width and length, and medial and lateral diagonals. The study indicated that there are no consistent, one-directional asymmetries in the right and left hoof solear aspects of horse forelimbs, although almost 1/3 of horses show some asymmetries. The hoof solear dimensions are similar in mares and geldings. The dimensions decrease as the size of the horse breed decreases. Purebred Arabian horses have a larger frog in the right hoof than other breeds. The dimensions increase with age in adult horses, apart from the hoof frog width and length.

## 1. Introduction

Since domestication, the selection criteria in horse breeding have undergone a fundamental shift from enhancing horse usability in transportation and agriculture to prioritising leisure and athletic performance under saddle [1]. However, the importance of sound and properly conformed hooves for healthy horses has remained constant. The conformation of the limbs, including the hooves, has a key influence on the health and mobility of the horse. Appropriate hoof balance is a significant factor to horses’ overall performance capability, supporting body weight, absorbing shocks, preventing slippage, and protecting the sensitive parts of the toes. From a clinical perspective, the hooves are decisive to horses’ overall health and welfare. The hard hoof capsule enables movement but is susceptible to many diseases and injuries that can cause pain, lameness and other health issues. Correct hoof conformation is essential for maintaining hoof balance, which significantly impacts the limb posture and movement biomechanics and reduces the risk of injury [2,3]. The scientific literature emphasises that any abnormality in hoof conformation can be associated with anomalies in the musculoskeletal system and result in locomotion disorders [4,5,6,7]. For example, Wilson et al. [8] showed that decreasing hoof width and length is associated with increased movement asymmetry.

Symmetry in body conformation, as well as the structure and posture of entire limbs, including hooves, all play an important role in horse performance [9]. Ducro et al. [10] found that uneven feet have a negative effect on athletic performance, mainly in jumping, and impact the duration of the horse’s competitive life. The term ‘uneven feet’ was coined to describe the occurrence of two differently shaped forefeet with regard to the hoof angle in a 27-week foal: (right − left)/(right + left) × 100. The foals with a value above mean were considered uneven [11]. Even a slight asymmetry can lead to modifications in growth patterns and hoof wear patterns, which consequently increase the risk of injury due to compensatory mechanisms. This is particularly relevant for sport horses, in which uneven limb loading can negatively affect stability and contribute to the development of chronic health problems [6,12]. Any defects in hoof shape may result in an uneven distribution of forces, which, in turn, can lead to overloading specific areas of the hoof and force abnormal movement patterns. Over time, these patterns can place excessive strain on muscles (e.g., interossei medii and flexoris digiti profundi) and flexor tendons [7]. As such, researchers focused on identifying specific variables of the hoof capsule. Wilson et al. [13] studied leisure horses and found asymmetry in the left–right morphometry of forelimb length, which is connected to asymmetry in hoof spread. In addition, the width of the left hoof was larger than that of the right hoof in 5.9% of horses more often than conversely. According to Leśniak et al. [12], the conformation of the bilateral hoof pair, also in leisure horses, is not always symmetrical, particularly in horses of a larger body mass and height. The left hoof capsule shape is broader and more acutely angled compared to the upright, boxy hoof on the right. In turn, a study on one-year-old Catalan Pyrenean horses showed homogeneity in size and a high level of symmetry in length, width and sole area of both front and hind hooves [14]. The difference with the above-mentioned leisure horses might have partly resulted from the fact that Catalan Pyrenean horses were reared outdoors and did not receive any trimming.

The process of trimming hooves is performed almost solely on the solear aspect. The knowledge of a possible solear aspect asymmetry may improve trimming. Most research to date has concerned the toe, wall, heel, coronary band structure and angles, etc. [4,6,15,16] while the solear aspect is less frequently examined [14,17]. The ‘solear aspect’ term is used in the study to indicate the whole bottom (inferior) side of the hoof including the ground surface of the hoof wall, white line, sole, frog and heels [18,19]. The solear aspect is the first to contact the ground and transfer ground reaction forces when it expands in consequence of loading. Among the solear aspect measurements, hoof width is the most strongly correlated between the fore and hind hooves, which shows that this dimension is more constant [20]. While an elongated hoof capsule, namely toe and heel length, is shortened by trimming [21], the hoof width is influenced the least. A slight decrease in the hoof width is mainly associated with rounding off the outer circumference of the hoof wall and also minimally results from the cone shape of the hoof capsule, whose upper parts have a smaller circumference [22].

Different functions and biomechanics of the fore and hind limbs cause the shape of the hoof capsule in these limbs to differ. The horse’s centre of gravity is located closer to the front of the trunk; therefore, the forelegs are more heavily loaded, equating to 58.7% in standing [20,23]. When jumping over a 1.3 m-high fence, the trailing forelimb lands with an initial force amounting to twice the body weight [24]. When the horse turns, the centrifugal force increases by 40% and presses down on a fore hoof, guided by the movement of the forelimbs [25]. These factors help explain why the hooves of the forelimbs are primarily considered in many studies [8,12,18], a consideration also employed in the current study.

Although the foregoing knowledge on the symmetry of hooves was gained by studying horses of various sexes, types, breeds, and ages, these factors were not taken into account in statistical analyses due to the usually modest sample size. The current study examines the symmetry of solear dimensions in the bilateral hooves of the forelimbs of horses across two sexes, four breeds and three age groups, measured with a calliper. The aim of the study was to determine and compare the solear aspect dimensions of bilateral hooves in forelimbs with regard to the sex, breed and age of horses. It was hypothesised that the bilateral hooves in forelimbs may not be symmetrical in the solear aspect in horses regarding these factors.

## 2. Materials and Methods

### 2.1. Horses

One hundred horses with correct limb posture and movement were qualified by an experimenter experienced in assessing horse conformation and a veterinary surgeon who evaluated the soundness of the locomotor system on the basis of movement observation. Horses that had any signs of lameness were excluded. The hooves of the horses were regularly trimmed (every six weeks). Mainly, the ground surface of the hoof wall was shortened by approximately 1 cm, whereas the sole and frog were saved and corrected when necessary. The hooves were measured between five days to two weeks after routine trimming. The hooves of the horses had not been shod for at least three years. The horses studied were differentiated with regard to three factors: sex, breed, and age. According to each factor, they were analysed in groups or as a whole. The population was composed of 76 mares and 24 geldings, comprising 32 Warmblood (WB), 36 Purebred Arabian (PA), 23 Polish Konik (PK), and 9 Felin Ponies (FP). The PK is a primitive breed, standing 130–140 cm at the withers [26]. The FP was founded based on various pony and little horse breeds [27]. The horses belonged to three age groups: 40 termed ‘young’ (4–8 years old), 38 ‘middle-aged’ (9–15 years old) and 22 ‘old’ (16–26 years old). The age groups were differentiated to make them as even as possible with regard to age ranges and number of horses in a group, despite the uneven number of horses available at each age for the study. It was assumed that until the age of 8 years old, the hooves are not worn, contrary to those after the age of 16 years old. The age information was recorded from passports. The horses were maintained in four breed facilities located in southeastern Poland and trimmed by four trimmers, one for each facility. The management systems in the facilities were the same: the animals were kept individually in box stalls, fed three times daily with oats and meadow hay, supplemented, and turned out into grassy paddocks for most of the day.

### 2.2. Measurement Data

**Figure 1:**
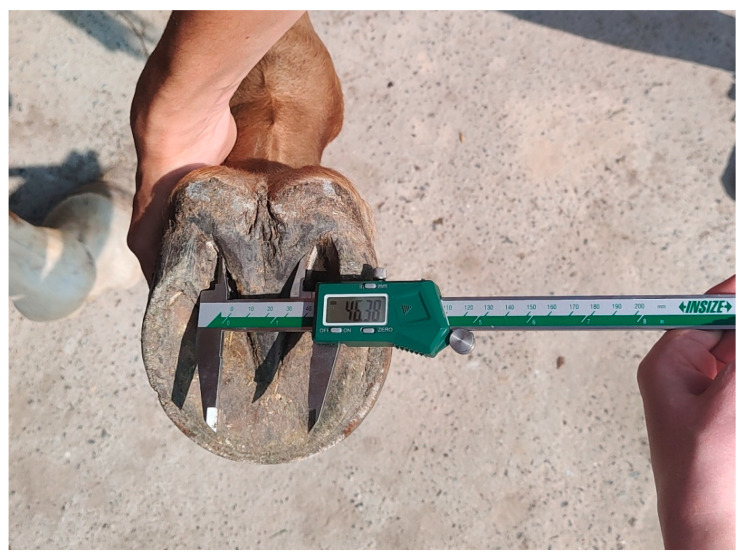
InSize calliper used in the study.

The solear aspect of the hoof was measured by one of the authors (WD) to ensure consistency in the method used. The method was first checked on four horses with the measurements repeated three times. Since the readings of the calliper were similar (± 1.5%), the next horses were examined once. For measuring, the hooves were cleaned with a hoofpick and a brush. The horses were restrained with a bridle and stood squarely on three feet on a flat, hard floor, with the measured foot elevated by a familiar caretaker. Ten to twelve horses daily were assessed with regard to the correctness of the locomotor system and examined (8:00–13:00). The measurements were carried out with an InSize calliper (Digital Callipers Standard Type, Code 1108-200, INSIZE CO., Ltd., Suzhou New District, Suzhou, China) with 1 mm accuracy (Figure 1).

**Figure 2:**
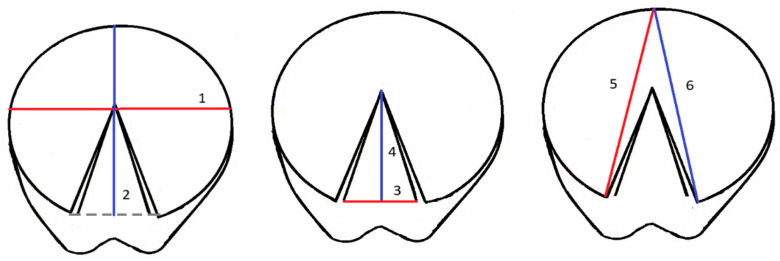
Biometric measurement of solear width (1—SW), solear length (2—SL), frog width (3—FW), frog length (4—FL), medial diagonal (5—MD) and lateral diagonal (6—LD) in the solear aspect of the hoof in the horse forelimbs.

The following dimensions were measured [8,18,20,28] (Figure 2):

- Hoof solear width (SW) measured at the widest part of the hoof;
- Hoof solear length (SL) measured from the centre of the toe to the centre of the heel buttress segment (in the palmar hoof line), fixed between heel buttress points (angles of the wall);
- Frog width (FW)—distance between edges of the frog at the heel buttress level;
- Frog length (FL)—distance between the frog apex and the centre of the heel buttress segment;
- Medial diagonal (MD)—distance between the centre of the toe and the medial heel buttress point;
- Lateral diagonal (LD)—distance between the centre of the toe and the lateral heel buttress point.

To make the SL, MD and LD measurements precise, at the beginning of the procedure, the toe centre was determined with the calliper. For this, the hoof width was divided into two, the value obtained was measured and the point was marked on the dorsal part of the hoof wall. The whole measurement of a horse lasted 15–20 min.

### 2.3. Statistical Analysis

To assess the hoof symmetry, the right and left hoof dimensions as well as a symmetry index were calculated for each dimension. The symmetry index compares the bilateral hooves of each horse [13]. The index was negative when the left hoof’s dimension was larger than that of the right hoof.

The statistical analysis of the data was performed using Statistica software (StatSoft), version 13.3. The Shapiro–Wilk and Lilliefors tests indicated that the data distribution was generally normal, and the Levene test confirmed homogeneous variances. Only the FL in the symmetry index distribution was not normal. The data were analysed according to the factors dividing the horses into groups: horse sex (mares, geldings), breed (WB, PA, PK, PN) and age (young, middle-aged, old). Since it was verified that no interactions occurred between the factors, ANOVA for main effects without interactions was used for normally distributed data. The significance of differences between means was analysed with the Tukey test (RIR) or Student’s *t*-test (depending on the number of means compared) in cases where significant factors were identified. Data for the FL symmetry index were tested with the Kruskal–Wallis test. The differences between the means were considered significant at *p* < 0.05 and highly significant at *p* < 0.01. Spearman correlation coefficients were determined.

To calculate the percentage of horses with asymmetric hooves, a threshold indicating the asymmetry in each horse was defined as the mean symmetry index for a horse group ± sd. Horses with right or left hoof dimensions higher than in the opposite hoof were considered together, apart from calculating the overall prevalence of left and right asymmetries in the whole population.

## 3. Results

The results of the significance test for the analysed factors are presented in Table 1. Breed as a factor was highly significant for all dimensions in right and left hooves (*p* < 0.01) as well as for FW (*p* < 0.01), FL (*p* < 0.01) and LD (*p* < 0.05) in the symmetry index. The age group of the horse was significant only for some dimensions not related to the frog (*p* < 0.01, *p* < 0.05), mainly in the left hooves. The sex of the horse was not significant for any of the dimensions.

**Figure 3:**
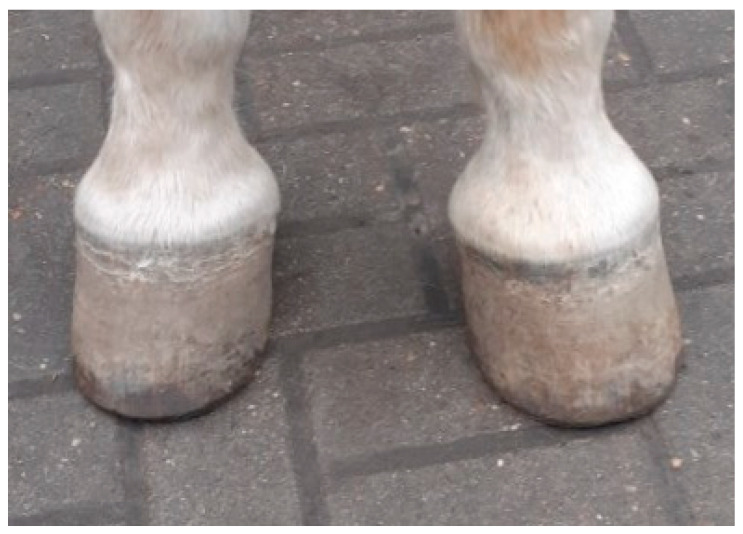
Dorsal view of hooves in forelimbs in Amin Pixie gelding at the age of 26 years. The left hoof is broader than the right hoof.

**Figure 4:**
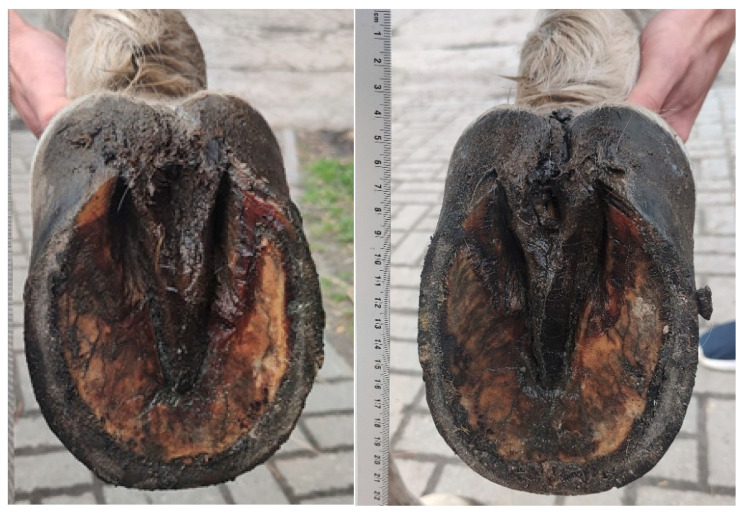
View of the solear aspect in the hooves of the forelimbs in Amin Pixie gelding at the age of 26 years. The width is larger in the left hoof than in the right hoof.

Table 2 and Table 3 present the SW and SL, respectively, depending on the factors considered. These dimensions were significantly higher (*p* < 0.01) in WB horses than in horses of other breeds, both in right and left hooves. In addition, wider (*p* < 0.01) and longer (*p* < 0.05) left hooves were found in horses in the old age group compared to the young group, but no such pattern was observed for right hooves. The symmetry index was positive for SW in all the analysed horse groups distinguished according to the factors studied, while for SL this index was positive for PA and young horses. Figure 3 and Figure 4 illustrate an example of asymmetric hooves in a horse.

In PA, the FW was higher (*p* < 0.05) in the right hoof than in the left hoof, whereas in PK, the opposite results were observed (Table 4). The variable was also significantly higher (*p* < 0.01) in WB compared to horses of other breeds, both for right and left hooves. Furthermore, the frog in the right hoof of PA was significantly wider than in PK, which was confirmed by the negative symmetry index (*p* < 0.01). The index was positive in all other cases and in total.

The FL was statistically higher (*p* < 0.05) in the right hoof than in the left hoof in PA horses (Table 5). When comparing breeds, FL was higher (*p* < 0.01) in WB than in other breeds, for both the right and left hooves. Furthermore, the frog in the left hoof was longer in PK than in FP (*p* < 0.05). The symmetry index differed significantly (*p* < 0.01) between PA and WB, as well as between PA and PK. Specifically, the right hooves had longer frogs than the left hooves in PA, whereas the opposite was observed in WB and PK. The symmetry index was only negative in four horse groups while it was positive in the whole sample.

Table 6 and Table 7 present the average MD and LD. These dimensions were greater in WB horses than in other breeds, both for left and right hooves (*p* < 0.01). In young horses, both diagonals in the left hoof and the LD in the right hoof were shorter than in old horses (*p* < 0.05). The symmetry index was negative in four horse groups for MD, while for LD this index was negative in seven horse groups and in the whole sample.

The percentage of horses in the whole population with the symmetry index above and below the mean (mean ± SD; Table 8) shows that asymmetries occurred in 23.0–32.0% of the horses, depending on the dimension with a slightly higher percentage for the right or left hoof.

**Figure 5:**
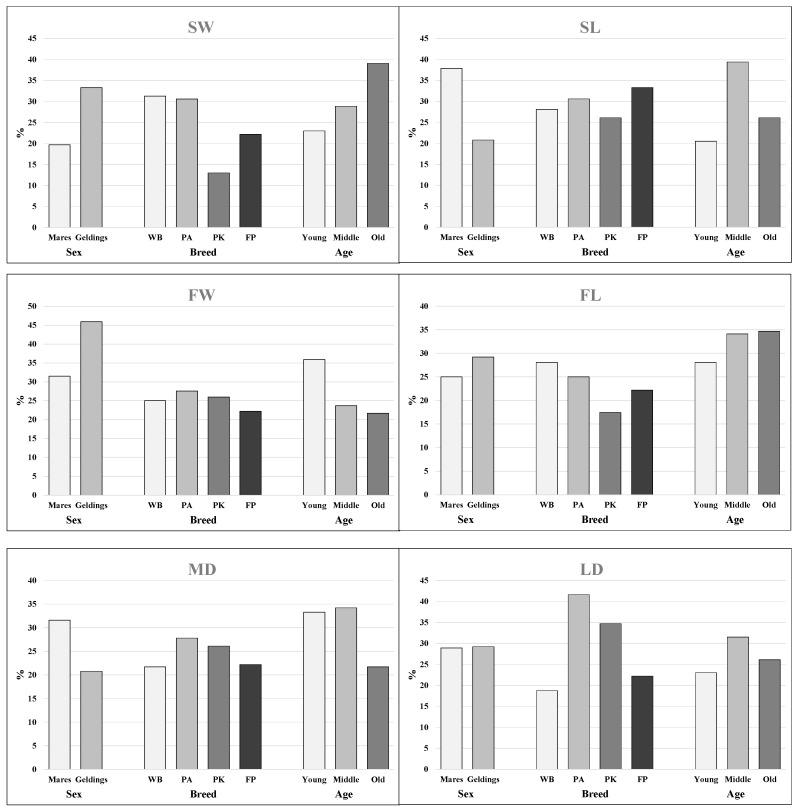
Percentage of horses with asymmetry in the dimensions of the right and left hooves in the whole population (n = 100) with regard to factors examined, SW—solear width, SL—solear length, FW—frog width, FL—frog length, MD—medial diagonal and LD—lateral diagonal, WB—Warmblood, PA—Purebred Arabian, PK—Polish Konik and FP—Felin Ponies.

The percentage of horses with asymmetries, irrespective of the fact whether the left or right hoof was of a higher dimension than the opposite, is illustrated in Figure 5. The percentage ranged from 13.0 (SW in PK) to 45.9 (FW in geldings). More geldings than mares can be noted in the case of SW, FW and FL. Considering the breeds, PK with SW and FL asymmetries were rare compared to other breeds. A low frequency of asymmetries in young horses can be observed in SW, SL, FL and LD.

**Figure 6:**
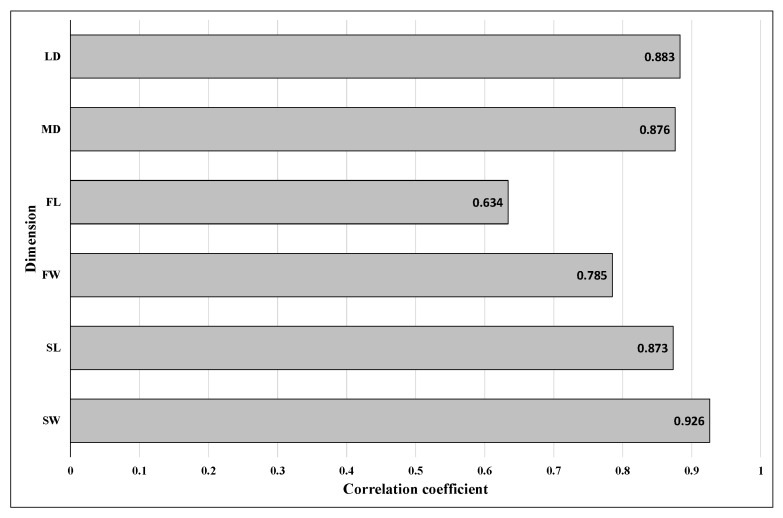
Correlation coefficients (R) in the dimensions between right and left hooves (*p* < 0.05; n = 100), SW—solear width, SL—solear length, FW—frog width, FL—frog length, MD—medial diagonal and LD—lateral diagonal.

Correlation coefficients in the dimensions between right and left hooves were high (*p* < 0.05; Figure 6). The highest coefficient was found for SW (R = 0.93), and the lowest coefficient was recorded for FL (R = 0.63).

## 4. Discussion

Morphological features of the foot and the relationship between limb posture and hoof conformation are crucial for proper gait and may reduce the risk of injury by appropriate trimming and therapeutic horseshoeing [6,18]. The study investigated whether there are any consistent differences between dimensions of the solear aspect of bilateral hooves in the forelimbs of various horses, specifically in relation to biological factors such as sex, breed, and age group. Direct reasons for an asymmetry may include foals adopting a wide-legged grazing stance with their necks short and limbs long at a young age [29]. Other reasons include injuries that lead to uneven loading of the bilateral limbs or continuous training and racing in one direction [30]. Foals should be observed to determine whether the hoof asymmetry occurs, as it cannot be excluded that the trait may be innate and result from prenatal disorders. Presumably, inexperienced trimmers may carry out the trimming, detrimentally increasing a slight defect.

Although the conformation of the entire hoof is considered when trimming, the interference is performed mainly on the solear aspect, particularly on the ground surface of the hoof wall. Humans may not be able to notice any asymmetries in the right and left feet since the hooves are trimmed one after another and are not visible simultaneously. Obviously, it is difficult to remember exactly the shape of a hoof trimmed when trimming the opposite one. It is commonly assumed that the solear aspects in bilateral hooves are symmetrical, although accurate relationships between the hooves are an open question.

Considering horse groups distinguished according to the factors analysed within the whole population, the current study found only three significant differences between the right and left hoof solear aspect mean dimensions out of a total of 54 differences (5.5%). These differences related solely to the frog variables: right hooves had wider frogs than left hooves in PA, but the opposite was true in PK. Additionally, frogs were longer in right hooves than in left hooves in PA. The mean differences between right and left hooves equalled 3.78 (FW)–7.89 mm (FL); however, along with the former statement, they do not seem to be sufficient to infer that there are any consistent, one-directional asymmetries in particular hoof dimensions. The symmetry index varied between positive and negative across horse groups, being often negative in SL and LD, which indicates larger left hoof dimensions. The total symmetry index, irrespective of the horse groups, was also negative for SL and LD. This can indicate that the left hooves tended to be longer than the right hooves. On the other hand, since the total SW, FW and FL symmetry indexes were positive, right hooves tended to be slightly wider than left hooves. Hence, considering the means in the population, very rare significant differences in average dimensions of right and left hooves and symmetry index, as well as a very slight tendency for longer and narrower left hooves, do not indicate a common phenomenon of consistent one-directional asymmetry of the hoof solear aspect in bilateral forelimbs in horses.

High correlations in dimensions of right and left hooves may also indicate a homogeneity of bilateral hooves and rare cases of asymmetric feet in individual horses, since a correlation describes the strength and direction of a linear relationship between two variables. Interestingly, the correlation coefficient for SW was the highest, which may confirm that this is the most constant and so characteristic parameter of the hoof [20].

However, contrary to the above-mentioned findings, it should be emphasised that the mean symmetry index, resulting from positive and negative values, tended to zero. Therefore, to deepen the analysis, the percentage of horses with asymmetries was determined. It was found that the asymmetries occurred in almost 1/3 of horses, depending on the dimension, with a lower frequency of LD asymmetry. This prevalence shows that hoof asymmetries are quite common in horses. In summary, although no directional asymmetry in the hoof solear aspect variables across the population was found, many horses do exhibit asymmetries.

As mentioned in Section 2, the identified asymmetries did not cause any diseases or disorders in the movement of the examined horses. Previously, Ducro et al. [29] found that the genetic correlation between uneven feet and performance in competition was negative but weak, suggesting that, despite many hypotheses, hoof asymmetries are not particularly harmful. However, it seems possible that asymmetrical shapes of bilateral hooves, especially SW, may cause abnormalities in the foot flight, i.e., the path the foot travels during a step, similar to horses that are toed out or toed in [31]. In turn, asymmetry in SL may differentiate the moment of breakover between bilateral feet leading to a subclinical lameness. According to Wiggers et al. [32], it is often hypothesised that asymmetric feet, like uneven feet, are important risk factors for the development of lameness. To correct the gait, the trimmer should make the hooves as symmetrical as possible [31].

Wilson et al. [13] studied the forelimbs in leisure horses of different breeds and types, showing that the capsule width tended to be larger in the left hoof which is inconsistent with the current results (Table 9). Studies by Wilson et al. [8] were more in agreement with the current study. In the Dutch Warmblood horse population, the prevalence of uneven feet was approximately 5.3% [29]. As mentioned, Parés-Casanova and Oosterlinck [14] documented a high level of symmetry in the length, width, and solear area of the front hooves. Based on the cited findings and the current results, it can be suggested that no consistent asymmetries in bilateral hoof solear measurements exist, despite frequent variations among individual horses.

Considering the factors studied, it is worth emphasising that sex does not influence the hoof solear dimensions. In consequence, there are no significant differences between bilateral hooves within mares or geldings. According to Lewis et al. [33], hoof growth in Morgan mares and geldings housed in a stable and turned out in a pasture during the night was similar. In turn, the current study reveals obvious differences in all of the hoof solear aspect dimensions in horses of various breeds. All dimensions were higher for WB horses, which are higher at the withers and heavier than other breeds studied. Among the other breeds, only PA had higher FW than PK in the right hoof, and the FL was higher in PK than in FP in the left hoof. These outcomes are consistent with the horses’ mean height at withers and body mass, which are larger in PA, smaller in PK, and the lowest in FP [26,34]. According to Leśniak et al. [12], the horse’s body mass is more influential on hoof morphology than withers height. Interestingly, the symmetry index significantly differed between PA and other breeds: PK with regard to FW as well as WB and FP considering FL. This fact may indicate that the frog in the right hoof is larger than in WB, PK and FP. It may be associated with uneven trimming of bilateral hooves in PA or with always leading these horses with the left hand during training for shows and exhibitions.

Regarding the age group effect, Ducro et al. [29] studied KWPN horses and found that the prevalence of uneven feet increases with age in both males and females. In the current study, bilateral hooves in horses of particular age groups did not reveal an asymmetry, although they tended to change with age. The symmetry displayed in particular age groups might have partly resulted from the uneven grouping described in Section 2. It should be noted that because the horses were four years old or older, their hoof capsules were mostly formed into the adult horse’s hoof shape [35]. Despite this, SW, SL, MD, and LD increased with age in the left hooves, and LD also increased in the right hooves. The FW and FL variables were unaffected. This suggests that the solear aspect of hoof dimensions continues to grow with age in adult horses, except for the frog width and length. This finding may be particularly important for horse owners when trimming hooves or fitting shoes. It is difficult to postulate why the frog dimensions do not change with the age of adult horses. It seems that the hoof biomechanics relying mainly on a large frog must be fully developed already in younger horses. The clinical importance of differences in hoof dimensions has been studied scarcely to date, and the direct relations between the dimensions and horses’ movement have not been specified [6,8].

Important limitations of the study include uneven, sometimes very small numbers of horses in groups divided according to the factors which could have influenced the results. Another limitation is the fact that different people trimmed the horses and might have done so in slightly different ways.

## 5. Conclusions

Overall, the findings of this study suggest that there are no consistent, one-directional asymmetries in the solear aspect of bilateral hooves in horse forelimbs. Contrary to this statement, almost 1/3 of horses exhibit asymmetries towards larger right or left hoof dimensions, which results from different directions of the differences and shows the importance of the issue. The hoof solear dimensions do not differ between mares and geldings. The dimensions decrease as the horse breed size decreases. Purebred Arabian horses have a larger frog in the right hoof than other breeds. Most variables increase with age in adult horses, except for the dimensions related to frog width and length. These findings expand the understanding of hoof conformation and may be of interest to those responsible for the care of horses’ hooves.

## Figures and Tables

**Table 1:** *p*-values for the effect of horse sex, breed and age group on the dimensions of right and left hooves and the symmetry index.

Variable	Factor	Right Hoof	Left Hoof	Symmetry Index
SW	Sex	0.615	0.874	0.272
	Breed	0.000 **	0.000 **	0.408
	Age group	0.249	0.040 *	0.079
SL	Sex	0.986	0.372	0.268
	Breed	0.000 **	0.000 **	0.207
	Age group	0.289	0.012 *	0.246
FW	Sex	0.751	0.266	0.266
	Breed	0.000 **	0.000 **	0.001 **
	Age group	0.631	0.783	0.492
FL	Sex	0.327	0.498	0.848
	Breed	0.000 **	0.000 **	0.000 **
	Age group	0.832	0.804	0.499
MD	Sex	0.794	0.632	0.336
	Breed	0.000 **	0.000 **	0.099
	Age group	0.054	0.007 **	0.757
LD	Sex	0.585	0.080	0.092
	Breed	0.000 **	0.000 **	0.013 *
	Age group	0.049 *	0.000 **	0.132

* *p* < 0.05, ** *p* < 0.01, SW—solear width, SL—solear length, FW—frog width, FL—frog length, MD—medial diagonal and LD—lateral diagonal.

**Table 2:** Solear width [SW; mm] of the horse’s right hoof and left hoof measured with a calliper, and symmetry index (difference between right and left hooves) with regard to the factors examined.

Factor		n	Right Hoof		Left Hoof		Symmetry Index	
			Mean	sd	Mean	sd	Mean	sd
Sex	Mares	76	113.67	11.80	112.57	12.16	1.11	5.24
	Geldings	24	127.54	15.19	125.29	14.43	2.25	5.70
Breed	WB	32	132.63 ^ABC^	12.49	131.25 ^DEF^	11.46	1.37	5.73
	PA	36	110.50 ^A^	7.67	108.58 ^D^	8.57	1.92	5.66
	PK	23	109.35 ^B^	4.37	108.70 ^E^	4.30	0.65	5.37
	FP	9	107.00 ^C^	6.76	105.89 ^F^	5.25	1.11	2.15
Age group	Young	39	113.87	11.36	111.26 ^G^	12.06	2.62	6.22
	Middle	38	115.92	14.63	115.42	13.71	0.50	4.60
	Old	23	124.09	14.86	123.35 ^G^	13.86	0.74	4.69
Total within a factor		100	117.00	13.95	115.62	13.79	1.38	5.35

n—number of observations, sd—standard deviation, WB—Warmblood, PA—Purebred Arabian, PK—Polish Konik and FP—Felin Ponies, differences between means marked with the same capitals (A, B, C, D, E, F, G in columns) are statistically significant at *p* < 0.01.

**Table 3:** Solear length [SL; mm] of the horse’s right hoof and left hoof measured with a calliper, and symmetry index (difference between right and left hooves) with regard to the factors examined.

Factor		n	Right Hoof		Left Hoof		Symmetry Index	
			Mean	sd	Mean	sd	Mean	sd
Sex	Mares	76	115.49	10.74	115.55	9.60	−0.07	5.88
	Geldings	24	123.79	12.73	125.42	11.47	−1.63	5.27
Breed	WB	32	129.00 ^ABC^	11.29	129.22 ^DEF^	9.68	−0.22	4.95
	PA	36	113.25 ^A^	6.27	112.83 ^D^	5.05	0.42	4.84
	PK	23	113.30 ^B^	7.04	114.22 ^E^	7.05	−0.91	7.96
	FP	9	104.11 ^C^	6.27	107.56 ^F^	8.16	−3.44	4.85
Age group	Young	39	114.31	10.37	113.90 ^a^	8.86	0.41	6.41
	Middle	38	119.08	12.19	119.55	11.14	−0.47	5.03
	Old	23	120.22	12.41	122.04 ^a^	11.71	−1.83	5.65
Total within a factor		100	117.48	11.74	117.92	10.88	−0.44	5.75

n—number of observations, sd—standard deviation, WB—Warmblood, PA—Purebred Arabian, PK—Polish Konik and FP—Felin Ponies, differences between means are statistically significant: marked with the same capitals (A, B, C, D, E, F in columns) at *p* < 0.01 and with the same small letters (a in columns) at *p* < 0.05.

**Table 4:** Frog width [FW; mm] of the horse’s right hoof and left hoof measured with a calliper, and symmetry index (difference between right and left hooves) with regard to the factors examined.

Factor		n	Right Hoof		Left Hoof		Symmetry Index	
			Mean	sd	Mean	sd	Mean	sd
Sex	Mares	76	41.50	10.69	40.97	11.49	0.53	7.45
	Geldings	24	48.46	14.15	47.13	13.97	1.33	9.57
Breed	WB	32	52.94 ^ABC^	13.38	52.94 ^EFG^	14.50	0.00	9.39
	PA	36	42.17 ^ADx^	7.35	38.14 ^Ex^	7.03	4.03 ^H^	6.14
	PK	23	32.87 ^BDy^	5.00	36.65 ^Fy^	7.30	−3.78 ^H^	6.24
	FP	9	38.78 ^C^	6.72	37.22 ^G^	7.55	1.56	7.83
Age group	Young	39	40.62	12.29	39.95	10.84	0.67	8.62
	Middle	38	44.32	11.92	43.63	13.30	0.68	6.83
	Old	23	45.61	10.91	44.74	12.88	0.87	8.86
Total within a factor		100	43.17	11.92	42.45	12.34	0.72	7.96

n—number of observations, sd—standard deviation, WB—Warmblood, PA—Purebred Arabian, PK—Polish Konik and FP—Felin Ponies, differences between means marked with the same capitals (A, B, C, D, E, F, G, H in columns) are statistically significant at *p* < 0.01, differences between means marked with the same small letters (x, y in rows) are statistically significant at *p* < 0.05.

**Table 5:** Frog length [FL; mm] of the horse’s right hoof and left hoof measured with a calliper, and symmetry index (difference between right and left hooves) with regard to the factors examined.

Factor		n	Right Hoof		Left Hoof		Symmetry Index	
			Mean	sd	Mean	sd	Mean	sd
Sex	Mares	76	69.97	9.76	67.09	11.34	2.88	10.61
	Geldings	24	72.54	13.47	74.21	13.05	−1.67	5.83
Breed	WB	32	78.22 ^ABC^	9.10	80.37 ^DEF^	8.31	−2.16 ^H^	6.47
	PA	36	72.17 ^Ax^	8.20	64.28 ^Dx^	8.59	7.89 ^GH^	9.25
	PK	23	64.52 ^B^	4.29	65.57 ^Ea^	9.07	−1.04	11.51
	FP	9	52.67 ^C^	7.23	54.00 ^Fa^	9.46	−1.33 ^G^	5.92
Age group	Young	39	69.05	10.68	66.67	12.00	2.38	12.65
	Middle	38	71.47	7.92	69.71	10.54	1.76	7.98
	Old	23	71.74	14.50	70.91	14.46	0.83	7.15
Total within a factor		100	70.59	10.75	68.80	12.10	1.79	9.85

n—number of observations, sd—standard deviation, WB—Warmblood, PA—Purebred Arabian, PK—Polish Konik and FP—Felin Ponies, differences between means are statistically significant: marked with the same capitals (A, B, C, D, E, F, G, H in columns) at *p* < 0.01 and with the same small letters (a in columns) at *p* < 0.05, differences between means marked with the same small letters (x in rows) are statistically significant at *p* < 0.05.

**Table 6:** Medial diagonal [MD; mm] of the horse’s right hoof and left hoof measured with a calliper, and symmetry index (difference between right and left hooves) with regard to the factors examined.

Factor		n	Right Hoof		Left Hoof		Symmetry Index	
			Mean	sd	Mean	sd	Mean	sd
Sex	Mares	76	113.88	11.57	114.75	11.00	−0.87	6.62
	Geldings	24	126.04	14.85	123.13	13.75	2.92	5.25
Breed	WB	32	131.66 ^ABC^	12.75	129.41 ^DEF^	11.80	2.25	5.51
	PA	36	109.67 ^A^	4.13	111.75 ^D^	5.60	−2.08	4.93
	PK	23	111.22 ^B^	7.82	112.35 ^E^	6.95	−1.13	8.68
	FP	9	106.78 ^C^	6.30	103.11 ^F^	3.10	3.67	5.72
Age group	Young	39	112.26	11.74	112.03 ^ab^	10.28	0.23	7.74
	Middle	38	118.68	13.32	119.47 ^a^	12.32	−0.79	5.17
	Old	23	121.39	14.44	120.30 ^b^	12.83	1.09	6.25
Total within a factor		100	116.80	13.41	11.76	12.19	0.04	6.50

n—number of observations, sd—standard deviation, WB—Warmblood, PA—Purebred Arabian, PK—Polish Konik and FP—Felin Ponies, differences between means are statistically significant: marked with the same capitals (A, B, C, D, E, F in columns) at *p* < 0.01 and with the same small letters (a, b in columns) at *p* < 0.05.

**Table 7:** Lateral diagonal [LD; mm] of the horse’s right hoof and left hoof measured with a calliper, and symmetry index (difference between right and left hooves) with regard to the factors examined.

Factor		n	Right Hoof		Left Hoof		Symmetry Index	
			Mean	sd	Mean	sd	Mean	sd
Sex	Mares	76	116.80	10.21	116.91	9.44	−0.11	5.58
	Geldings	24	126.62	13.54	128.50	13.51	−1.88	5.82
Breed	WB	32	131.09 ^ABC^	11.40	131.25 ^DEF^	12.02	−0.16	4.47
	PA	36	114.64 ^A^	5.76	113.69 ^D^	4.48	0.94	4.57
	PK	23	113.83 ^B^	8.00	116.57 ^E^	7.46	−2.74	7.76
	FP	9	108.44 ^C^	5.27	110.56 ^F^	6.88	−2.11	5.88
Age group	Young	39	114.85 ^ab^	9.60	114.74 ^cd^	8.17	0.10	6.27
	Middle	38	121.18 ^a^	11.97	122.08 ^c^	11.97	−0.89	5.51
	Old	23	123.13 ^b^	13.05	124.13 ^d^	13.25	−1.00	4.88
Total within a factor		100	119.16	11.81	119.69	11.60	−0.53	5.66

n—number of observations, sd—standard deviation, WB—Warmblood, PA—Purebred Arabian, PK—Polish Konik and FP—Felin Ponies, differences between means are statistically significant: marked with the same capitals (A, B, C, D, E, F in columns) at *p* < 0.01 and with the same small letters (a, b, c, d in columns) at *p* < 0.05.

**Table 8:** The percentage of horses in the whole population (n = 100) with the symmetry index (difference between right and left hooves) above (dimension in right hoof higher) and below (dimension in left hoof higher) the mean (mean ± SD).

Dimension	Dimension in Right Hoof Higher	Dimension in Left Hoof Higher	Overall Percentage of Horses with Asymmetries
SW	11.0	18.0	29.0
SL	14.0	15.0	29.0
FW	13.0	16.0	29.0
FL	14.0	10.0	24.0
MD	16.0	16.0	32.0
LD	14.0	9.0	23.0

SW—solear width, SL—solear length, FW—frog width, FL—frog length, MD—medial diagonal and LD—lateral diagonal.

**Table 9:** The width of left and right hoof capsules [mm] in horses according to different sources.

Number of Horses	Right Hoof Capsule Width	Left Hoof Capsule Width	References
34	144.1	145.2	Wilson et al. [13]
43	129.3	122.3	Wilson et al. [8]
100	117.0	115.6	Current study

## Data Availability

The data presented in this study are available on request from the corresponding author.
